# Short-Term Memory Improvement After Simultaneous Interpretation Training

**DOI:** 10.1007/s41465-017-0011-x

**Published:** 2017-02-27

**Authors:** Laura Babcock, Mariagrazia Capizzi, Sandra Arbula, Antonino Vallesi

**Affiliations:** 1 Department of Neuroscience, Karolinska Institutet, Retzius väg 8, 171 77 Stockholm, Sweden; 2 Department of Neurosciences, University of Padova, via Giustiniani 5, 35128 Padova, Italy; 3 Fondazione Ospedale San Camillo, IRCCS, via Alberoni 70, 30126 Lido-Venice, Italy

**Keywords:** Simultaneous interpretation, Short-term memory, Executive functions, Training, Life-experience

## Abstract

Simultaneous interpretation (SI) is a cognitively demanding process that has been associated with enhanced memory and executive functions. It is unclear, however, if the previously evidenced interpreter advantages are developed through training and/or experience with SI or rather represent inherent characteristics that allow success in the field. The present study aimed to disentangle these possibilities through a longitudinal examination of students earning a Master of Conference Interpreting and two control populations. The students were tested at the beginning and end of their programs on measures of memory and executive functioning that have previously demonstrated an interpreter advantage. The results revealed no inherent advantage among the students of interpretation. However, an SI training-specific advantage was revealed in verbal short-term memory; the students of interpretation, but not the two control groups, showed a gain between the testing sessions. This controlled longitudinal study demonstrates that training in simultaneous interpretation is associated with cognitive changes.

**Electronic supplementary material:**

The online version of this article (doi:10.1007/s41465-017-0011-x) contains supplementary material, which is available to authorized users.

## Introduction

Simultaneous interpretation (SI) is potentially one of the most cognitively demanding tasks of human cognition. It requires an individual to simultaneously comprehend a stream of auditory material in one language while producing the same content in another language. It is thus unsurprising that individuals who have mastered this skill, that is, professional interpreters, have shown advantages on several measures of memory and executive functioning. Advantages in both short-term and working memory have been widely evidenced (e.g., Babcock and Vallesi 2017; Bajo et al. 2000; Christoffels et al. 2006; Padilla et al. 1995). Additionally, interpreters have been shown to be less affected by articulatory suppression, that is, the process of blocking maintenance of information by repetition of unrelated speech, during a memorization task (e.g., Bajo et al. 2000; Padilla et al. 1995; Yudes et al. 2012). More recently, advantages among interpreters have been seen in the orienting attentional network (Morales et al. 2015), in the coordination of multiple tasks in a dual-task paradigm (Becker et al. 2016; Strobach et al. 2015), and in sustained control in task-switching paradigms (Babcock and Vallesi 2017; Becker et al. 2016). In all cases, these advantages were found in comparison to bilinguals and multilinguals, suggesting they are not simply due to a “bilingual advantage” (if one exists, see Paap et al. (2015) for a discussion). Indeed, while bilingualism is a necessary prerequisite for the process of interpretation, it is not sufficient. Instead, individuals must choose to acquire the skill of interpretation, usually through intensive and specific training programs.

This begs the question, what is the provenance of the previously found interpreter advantages. These abilities may be acquired through specific training and/or later experience with simultaneous interpretation. This understanding is supported by studies which show that training in specific skills, such as meditation and video game playing, can lead to cognitive changes (e.g., Chiesa et al. 2011; Green and Bavelier 2003; Zeidan et al. 2010; but see Boot et al. 2011). Additionally, targeted training in the cognitive processes of shifting and updating has resulted in cognitive changes, particularly when complex tasks using multiple information streams and executive functions are employed (e.g., Strobach et al. 2014). Thus, training in simultaneous interpretation, which places high demands on memory and other executive control processes, may lead to the enhancement of specific processes. Indeed, a recent longitudinal functional magnetic resonance imaging (fMRI) study found a decrease in activation during interpretation in the caudate nuclei, which support executive functions, after training (Hervais-Adelman et al. 2015). If SI training is also responsible for cognitive changes, it could represent an opportunity to understand how an extraordinarily demanding profession may shape human cognition naturalistically.

Alternatively, the interpreter advantages may be due to inherent characteristics that enable success in the field. Simultaneous interpretation is a highly selective career path. Beyond proficiency in a minimum of two or three languages, individuals must complete and pass a series of qualification exams to enter both training programs and professional associations. Thus, the individuals who consider and ultimately succeed in becoming professional interpreters may be those who naturally have the required abilities. Understanding the cognitive profiles of successful candidates may then prove useful in the selection process. This could be of critical importance as interpretation is one of the fastest growing professions (e.g., United States Bureau of Labor Statistics 2015).

A handful of studies have attempted to examine the influence of SI training and experience on cognitive control by comparing groups with different levels of SI experience. Padilla et al. (1995) examined professional interpreters in comparison with two groups of interpretation students (before and after courses on simultaneous interpretation) and non-interpreter controls. The professional interpreters showed better performance than the other three groups on tests of verbal short-term and working memory (digit span and reading span, respectively). Additionally, the professionals were unhindered by articulatory suppression in a verbal recall task, while the remaining three groups all showed a decrement in performance. These results suggest that the memory and articulatory suppression advantages are not inherent, but rather acquired through experience with simultaneous interpretation.

More recent studies, however, have demonstrated some support for inherent differences in verbal working memory and articulatory suppression. Yudes et al. (2012) reported similar performance among professional interpreters and students of interpretation on a reading span task with both groups performing better than a control group of monolingual undergraduates. A study by Liu and colleagues also found no difference in verbal working memory (tested with a listening span task) between professional interpreters and two groups of interpretation students (Liu et al. 2004). Additionally, a study assessing students of interpretation at the close of their first and second years and a control group of bilingual students found that both groups of interpretation students outperformed the control group on a reading span task (Tzou et al. 2012). Finally, Köpke and Nespoulous (2006), which examined professional interpreters, students of interpretation, bilingual professionals, and monolingual students, reported that the students of interpretation outperformed the two control groups on a listening span task, while the professional interpreters did not differ from any of the groups. A similar pattern of effects was evidenced on a verbal recall task under articulatory suppression in the same study. This pattern of similar performance among professionals and students which is higher than control groups would indicate that these skills are either inherent or developed after very little training.

Yet three studies also provide evidence for a role of training in the form of enhanced performance with increased training and/or experience. The study by Yudes and colleagues found that on a verbal recall task with articulatory suppression the professional interpreters showed a decrease in recall only on the most difficult of four conditions (Yudes et al. 2012). The students, on the other hand, were hindered in two of the conditions and the monolinguals in all four conditions. Dong and Xie (2014) reported a similar pattern on the Wisconsin Card Sorting Task (WCST), on which students with 3 years of interpreting classes completed more categories than students with 1 year of courses who in turn outperformed non-interpreting students. Finally, Tzou and colleagues reported that second year students performed better than the bilingual controls on a short-term memory task (digit span), while the first year students did not differ from either group (Tzou et al. 2012).

Finally, a few studies have also reported some null and inconclusive results. Köpke and Nespoulous (2006) found no differences across their four groups (professional interpreters, students of interpretation, bilingual professionals, and monolingual students) on tasks of verbal short-term memory (digit span and word span). That study also examined the color-word Stroop task using English, French, and bilingual versions. The students of interpretation provided more correct responses in 45 s than the professional interpreters and bilinguals on one of the bilingual versions; no differences were seen on the other three versions. Two additional studies examined students of interpretation on tasks of conflict resolution. Dong and Xie (2014) compared students of interpretation to students of other subjects on the flanker task and found no difference in performance. Similarly, Woumans and colleagues found no differences between students of interpretation and balanced bilinguals on the Attention Network Test (ANT) and Simon task (Woumans et al. 2015).

These studies must be interpreted with caution, though, as they all employed a cross-sectional design. The comparison of professionals to students carries a built-in age difference that may have contributed to the effects. In particular, the advantages for students seen in the study by Köpke and Nespoulous may be explained by age-related changes in cognition.

Longitudinal studies of individuals learning to interpret offer a better method to explore the origin of the interpreter advantages (Abutalebi and Green 2007; Green et al. 2014; Macnamara 2012). We are aware of only two longitudinal studies that investigated changes in memory and cognitive control in students of simultaneous interpretation. Macnamara and Conway (2014) examined students of American Sign Language (ASL) interpretation during their first and fourth semesters of training. These students evidenced improvements on a task-switching paradigm, the Wisconsin Card Sorting Test, number-letter sequencing, and backward digit span tasks; however, no change was seen on reading and operation span tasks. It should be noted, however, that signed language interpretation may be qualitatively different than interpretation between two spoken languages. As there is only one spoken language stream in signed language interpretation, the level of interference between the languages is likely lower. Indeed, evidence from studies of bimodal bilinguals (one spoken language and one signed language) suggests that these individuals do not exhibit the same level of cognitive enhancements that unimodal bilinguals seem to show (e.g., Emmorey et al. 2008). Thus, the results of this longitudinal study may not generalize to interpretation between two spoken languages. Additionally, and more critically, a control group was not included in this longitudinal design. Therefore, it is not clear if the improvements seen were due to training in interpretation or rather to repetition of the task (e.g., learning effects). The same is true of the second longitudinal study on training in SI. Chmiel (2016) examined the English reading span abilities of Polish conference interpretation students in the first and last months of their 2-year Master’s program. Unlike the previous study, a significant improvement in reading span was found; further, before training, the students performed similarly to non-interpreter bilinguals and after training to professional interpreters. However, as neither of these latter groups were examined longitudinally, the improvement cannot be pinpointed to interpretation training. This is particularly germane given that the reading span task was conducted in their second language, which certainly improved over the 2 years. As memory span in a second language may be associated with proficiency in that language (e.g., Service et al. 2002), the choice of language may explain the improvement seen in Chmiel (2016) and the discord with Macnamara and Conway (2014). Thus, the current literature provides some evidence for improvements in memory and switching abilities over the course of training in simultaneous interpretation, but it has not sufficiently isolated SI training as the source.

The present study aimed to understand whether training in simultaneous interpretation or rather inherent abilities are responsible for the benefits in cognition seen among professional interpreters. To this end, we conducted a longitudinal study of students pursuing a Master in Conference Interpreting. As mentioned above, however, longitudinal studies have a built-in confound due to the passage of time and repetition of tasks. To address this confound, we compared this group of interest to two control populations. The first were students earning a Master in Translation. These students had the same educational background as the students of interpretation, as a Triennale (the Italian equivalent of a Bachelor’s degree) in Languages is required for matriculation in both programs.^1^ Additionally, similar to the students of interpretation, the Translation students spoke multiple languages and were engaged in language improvement and high-level language usage during the intervening period. Critically, though, the students of translation did not learn the simultaneous interpretation skill. Thus, the comparison of these two groups can specifically target training in simultaneous interpretation. The second control group consisted of individuals pursuing advanced studies in non-language fields. The inclusion of this group allowed us to tease apart the potential effects of increased multilingualism from the general effects of time and task repetition.

We focused our examination on processes that have previously evidenced interpreter advantages and are posited to be taxed during simultaneous interpretation. Primary among these were short-term and working memory which are necessary to store content between input and output and rehearse pre-output reformulations; they have also regularly revealed an advantage among professional interpreters (e.g., Babcock and Vallesi 2017; Bajo et al. 2000; Christoffels et al. 2006; Padilla et al. 1995; Signorelli et al. 2012; Stavrakaki et al. 2012; Yudes et al. 2011; Yudes et al. 2012). To reduce these high memory demands, interpreters also employ a number of strategies including the use of contextual cues to predict future input (e.g., Seeber and Kerzel 2011), which is potentially related to a previously found advantage in orienting attention (Morales et al. 2015). Finally, interpreters must maintain active two language systems, which may account for the advantages found in sustained control (Babcock and Vallesi 2017; Becker et al. 2016). Thus, we examined performance on tasks that measure these abilities, employing a battery of memory and executive functioning tasks that we have previously used with professional interpreters (Babcock and Vallesi 2017). The results of that study suggested that differences between the groups may be expected in verbal short-term and working memory, spatial short-term memory, and sustained control (measured with the mixing cost in the task-switching paradigm). Better performance among the students of interpretation compared to the two control groups before training began would provide evidence that interpreters possess some inherent abilities. Alternatively, an increase in performance after training among the students of interpretation, but not the control groups, would suggest that the advantages seen in professional interpreters are due to their specific training. Such differences would additionally provide evidence that the enhancements in memory and executive functioning seen in targeted laboratory training could extend to an ecologically valid training. Finally, a lack of differences between the groups at either time point may suggest a role of SI experience beyond the 2-year training period. Importantly, it should be noted that the abilities are not intrinsically linked, thus some abilities may be inherent and others trained.

## Methods

### Participants

One hundred twenty-seven students attending universities located in northeastern Italy (Trieste, Padova, and Forlì) were initially examined, 92 of whom participated in the study longitudinally.^2^ The students were recruited to form three groups based on their field of study. The first group consisted of 55 students (46 females) pursuing a Master degree in Conference Interpreting at the University of Trieste and the University of Bologna, Forlì campus. These students were tested at the start of their Master’s program and 47 of them (38 females) at the conclusion of the program as well (mean time between phases = 19.4 months, SD = 2.8, range 14.8-24.4). The second group consisted of 21 students (18 females) earning a Master degree in Translation at the University of Trieste. As with the Interpretation students, these students were tested at the start of their Master’s program and ten^3^ (eight females) returned for testing at the conclusion of their program (mean time between phases = 24.1 months, SD = 1.0, range 22.9–25.7). The third group of participants was composed of 51 students (32 females) of non-language subjects (chemistry, engineering, functional genomics, law, medicine, neuroscience, pharmacology, physics, and psychology) from the Universities of Trieste and Padova, the majority of whom were earning a Master degree. Thirty-five of these participants (20 females) were tested twice with approximately the same intervening time between the sessions as the other two groups (mean time between phases = 21.0 months, SD = 2.5, range 16.3–26.2). The majority of these students were tested at the start and conclusion of their Master’s program; those who were tested at different time points were, however, full-time students during the intervening 2 years. The original three groups were matched in terms of age (*p* = .390), years of education (*p* = .557), and socioeconomic status (measured as mother’s years of education; *p* = .116). This was true also among the subset that participated longitudinally (age: *p* = .153; education: *p* = .403; socioeconomic status: *p* = .319); however, differences in the intervening time were present (*p* < .001) and considered in the statistical analyses. All participants reported normal color vision and had no known neurological or psychiatric problems. Participants gave written informed consent and were compensated for their time. The study was approved by the ethical committee of the Scuola Internazionale Superiore di Studi Avanzati, Trieste, and the Bioethical Committee of the Azienda Ospedaliera di Padova.

### Tasks and Procedure

Participants were tested individually on tests of short-term memory, tests of working memory, the ANT, and a task-switching paradigm at phase 1, and again at phase 2 for those who participated longitudinally. Additionally, participants completed an in-house language history questionnaire at phase 1, which was updated at phase 2.

#### Language History Questionnaire

Participants were asked to provide information about all of the languages they knew and/or studied. For each language, they were asked to provide a self-rating in the areas of reading, writing, speaking, and understanding on a five-point Likert scale. At phase 1, they were also asked to evaluate how often they switched between languages within a conversation at home/with friends and at school using a five-point Likert scale. Finally, they were asked to quantify, using percentages, how much they used each of their languages at home/with friends and at school. At the second phase, the participants additionally completed a questionnaire developed to identify functional fluency in non-native languages. Functional fluency was operationalized as a B2 level or above in the Common European Framework of Reference for Languages (CEF). The questionnaire asked participants to give their CEF level and respond to eight yes-or-no questions that targeted the B1-B2 border (see Appendix for questionnaire items). The questionnaire contained two items for each of the four abilities (reading, writing, speaking, and oral comprehension), one item focused on academic usage and the other on personal usage. Participants were considered functionally fluent in languages for which they responded yes to seven or eight items. A one-way ANOVA conducted on the number of functional languages at phase 2 revealed a significant difference between the groups (*F*(2,83) = 61.252, *p* < .001). Post hoc Tukey tests revealed significant differences between the Non-language students and the Interpretation and Translation students (*p* < .001 for both comparisons), but no difference between the latter two groups (*p* = .944).

To further examine the equivalence of language proficiency between the Interpretation and Translation students, we compared their self-ratings at phase 1 (on the original groups and longitudinal subsets) and at phase 2. However, given that participants were asked to report all languages they had studied, even for brief periods, an average across all non-native languages would not have been an accurate representation of their abilities. Instead, the self-ratings were averaged across the two or three languages each participant was studying in their Master’s program. These comparisons identified an advantage for Interpretation students in speaking ability at phase 1 (on original groups: *t*(66) = 3.217, *p* = .002; on longitudinal groups: *t*(47) = 3.606, *p* = .001), but there were no other differences between the groups at either phase 1 or phase 2 (*p*s ≥ .191; see Table 1 and Supplementary materials for values).

**Table 1:** Biographical and language characteristics of the longitudinal participants by group

	Interpretation students (*N* = 47)	Translation students (*N* = 10)	Non-language students (*N* = 35)
Age at phase 1 (in years)	22.8 (1.8)	24.2 (3.6)	23.0 (1.9)
Years of education at phase 1	16.3 (1.1)	16.5 (0.7)	16.7 (1.7)
Mother’s years of education^a^	13.6 (3.8)	13.1 (3.2)	12.4 (2.9)
Number of functional languages at phase 2^b^	3.3 (0.8)	3.2 (0.6)	1.6 (0.5)
Phase 1 averaged reading level^c,d^	4.2 (0.4)	4.2 (0.4)	
Phase 1 averaged writing level^c,d^	3.7 (0.4)	3.5 (0.5)	
Phase 1 averaged speaking level^c,d^	3.8 (0.5)	3.2 (0.5)	
Phase 1 averaged understanding level^c,d^	4.1 (0.4)	4.1 (0.5)	
Phase 2 averaged reading level^c^	4.5 (0.4)	4.5 (0.5)	
Phase 2 averaged writing level^c^	3.8 (0.5)	4.0 (0.4)	
Phase 2 averaged speaking level^c^	4.0 (0.5)	3.8 (0.4)	
Phase 2 averaged understanding level^c^	4.3 (0.4)	4.3 (0.4)	
Switching frequency at home/with friends^e^	3.4 (1.3)	3.3 (1.2)	
Switching frequency at school^e^	3.9 (1.0)	3.7 (1.1)	
Number of languages used at home/with friends^f^	3.0 (1.1)	2.3 (0.8)	
Number of languages used at school^f^	3.7 (0.9)	3.3 (0.7)	

Values in parentheses are standard deviations

^a^Data not available for one Non-language student

^b^Data not available for six Non-language students

^c^These values were averaged across the two or three languages each participant studied as part of their Master’s program

^d^Data were not available for eight Interpretation students

^e^Data were not available for eleven Interpretation students

^f^Data were not available for five Interpretation students

Finally, to ensure similar language usage between the Interpretation and Translation students, we examined their switching frequency and number of languages used (calculated from the percent usage questionnaire counting languages assigned at least 5%). The two groups did not differ in switching frequency either at home/with friends (on original groups: *p* = .953; on longitudinal groups: *p* = .748) or at school (on original groups: *p* = .652; on longitudinal groups: *p* = .516). There was also no difference in the number of languages used at school (on original groups: *p* = .496; on longitudinal groups: *p* = .191); however, the Interpretation students used marginally more languages at home/with friends (on original groups: *t*(69) = 1.827, *p* = .072; on longitudinal groups: *t*(50) = 1.815, *p* = .076), though both groups on average used more than two languages (see Table 1 and Supplementary materials for values).

#### Short-Term Memory Tests

Computerized versions of the letter span and matrix span tasks (Kane et al. 2004) were used to assess short-term memory (STM) in the verbal and spatial domains, respectively. These two tasks followed the same format; a sequence of items of variable length was presented and the participants were asked to recall the items in the order they were presented at the end of each sequence. In the letter span task, the to-be-recalled items were 12 consonants. The items in the matrix span task consisted of a 4 × 4 grid with one square colored red; the position of the red square was the to-be-recalled item. The performance measure in each of these tasks was the sum of items in perfectly recalled sequences (or the absolute score as denoted by Engle et al. 1999). The reader is referred to Babcock and Vallesi (2017) for further task details.

#### Working Memory Tests

Working memory (WM) in the verbal and spatial domains was assessed using the automated operation span task and the automated symmetry span task, respectively (Unsworth et al. 2005). The format of these two tasks was identical. As in the STM tasks, participants were asked to recall sequences of items of varying length; however, prior to each item of the sequence an intervening task was presented. Participants received training on each task component separately, as well as together, before completing the test sequences. The intervening task consisted of an arithmetic operation (e.g., (2 × 6) − 4 = ?) in the operation span task and a symmetry judgment in the symmetry span task. Two performance measures were recorded: the number of items in perfectly recalled sequences and the number of errors on the intervening task. This latter measure included incorrect responses and responses that required a much longer than average response time (calculated during the intervening task training). Further details can be found in Babcock and Vallesi (2017).

#### ANT

This task was adapted from Costa and colleagues (Costa et al. 2009, 2008) and identical to that used in our previous study except for the language of the instructions (Babcock and Vallesi 2017). Stimuli consisted of five arrows located either above or below a central fixation cross. The four outer arrows pointed in a uniform direction, the central arrow, however, pointed in either the same direction as the others (congruent trials) or the opposite direction (incongruent trials). A congruent central arrow was shown in 75% of the trials and an incongruent central arrow in the remaining 25%. Participants were asked to indicate the direction of the central arrow as quickly and accurately as possible. Prior to each stimulus, a cue was presented which belonged to one of four types: no cue, central cue, double cue, and spatial cue. No cue trials presented the fixation cross throughout the cue period. The central cue replaced the fixation cross with an asterisk. The double cue presented asterisks at both potential locations of the central arrow (above and below the fixation cross) in addition to the fixation cross. The spatial cue showed the fixation cross plus an asterisk at the location where the central arrow would occur (either above or below the fixation cross). Specific details about the trials can be found in Babcock and Vallesi (2017).

This task allowed for the computation of three measures which examine the executive function, alerting, and orienting networks (Fan et al. 2002). The conflict effect, which is the difference in accuracy or response time (RT) between congruent and incongruent trials, provided a gauge of the executive function network. The alerting network was measured by the difference between trials with no cue and those with a double cue. Finally, the difference between trials with a spatial cue and trials with a central cue provided a measure of the orienting network.

#### Task-Switching Paradigm

The paradigm was a modified version of the task used in Rubin and Meiran (2005) and a shortened version of the task used in our previous study (Babcock and Vallesi 2017). Participants viewed red and blue hearts and stars presented individually on a white background and were asked to respond to either the color or the shape of the stimulus. A visual cue located above the stimulus indicated which task should be completed. Graphic cues were used to limit linguistic coding of the cue and stimuli. To allow examination of potential differences in endogenous and exogenous task reconfiguration (Meiran 1996; Rogers and Monsell 1995), two cue-to-target intervals (CTI) were employed (100 and 1000 ms). Participants were asked to make a choice response to each stimulus using two response buttons. The four possible response-to-button mappings (left: red/heart, right: blue/star; left: red/star, right: blue/heart; left: blue/heart, right: red/star; left: blue/star, right: red/heart) were counterbalanced across participants.

The paradigm included three blocks of trials. Blocks 1 and 2 were single-task blocks which required only one judgment (color or shape) for the entire block. The order of the judgments in the single-task blocks was counterbalanced across participants. Block 3 was a mixed-task block in which half of the trials required a color judgment and the other half a shape judgment. On half of the trials, the task to be completed was the same as on the previous trial (a repetition trial), while on the other half the task was different than on the previous trial (a switch trial). For further task details, the reader is referred to Babcock and Vallesi (2017).

From the three trial types presented in this paradigm (switch, repetition, and single-task) two main comparisons can be drawn. The comparison of the switch and repetition trials in the mixed-task block, referred to as the switching cost, provided a measure of the transient control needed to switch tasks. The difference between the repetition trials in the mixed-task block and trials in the single-task blocks, termed the mixing cost, was informative about the sustained control needed in the mixed-task block.

#### Data Analysis

The data analyses aimed to examine both inherent differences between the groups as well as differences that emerged after training. To address the former, the full sample of participants was considered, while for the latter only those who participated longitudinally were included. To determine if inherent differences in memory existed between the three student groups, one-way ANOVAs were performed on the score at phase 1 for each of the four memory tests and the number of errors on the two working memory tests. To examine the influence of SI training on memory, mixed-effects ANOVAs with phase as a within-subjects factor and group as a between-subjects factor were computed on the memory scores and working memory errors. Post hoc Tukey tests were used to investigate significant results in these analyses, unless otherwise stated.

Data from the ANT and task-switching paradigm were analyzed using a common procedure. Participant outliers based on accuracy rate (more than 3 interquartile ranges below the 1st quartile) were identified and removed on a task-by-task basis. Kruskal-Wallis tests were used to compare the accuracy data of the groups given that these data were not normally distributed. The first trial in each block was not considered for all analyses on accuracy. For the RT data, the first trial in each block and error trials were excluded. Additionally, for each participant, trials with an RT more than 3 standard deviations (SD) from their individual task mean were excluded (block-type mean was used for the task-switching paradigm). Finally, trials following an error were excluded to avoid post-error slowing confounds (Burns 1965). To investigate inherent differences between the groups, one-way ANOVAs were computed on the overall RT, conflict effect, alerting effect, and orienting effect on the ANT and on the block-type RTs, switching cost, and mixing cost on the task-switching paradigm at phase 1. Mixed-effects ANOVAs which included phase and trial type as within-subjects factors and group as a between-subjects factor were employed to examine training-related differences. Significant results were examined with post hoc tests, Bonferroni-corrected for the Kruskal-Wallis tests, and Tukey tests for the ANOVAs.

## Results

### Short-Term Memory

**Fig. 1:**
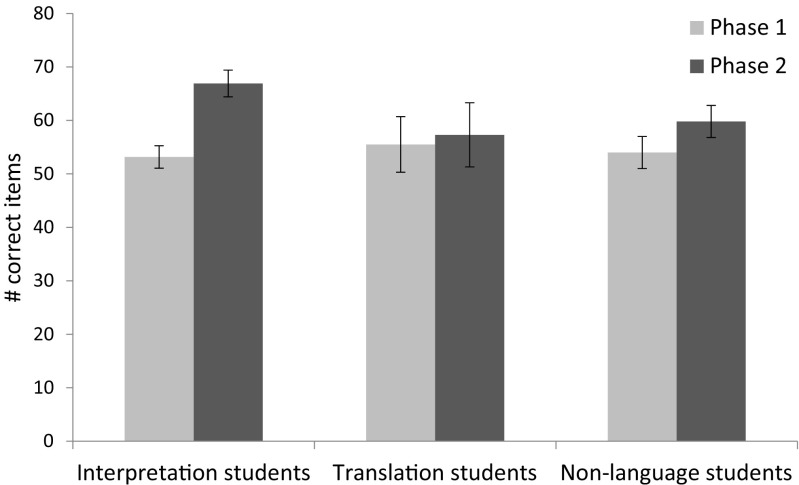
Performance on verbal short-term memory by group and phase. *Error bars* represent standard errors of the mean

The letter span task revealed no differences between the groups at phase 1 (*p* = .345). Further, there was no main effect of group across the phases (*p* = .563). There was, however, an interaction between group and phase (*F*(2,89) = 3.662, *p* = .030, *η*_*p*_^2^ = .076; see Fig. 1 and Table 2 for values), as well as a main effect of phase (*F*(1,89) = 11.846, *p* = .001, *η*_*p*_^2^ = .117). Post hoc *t* tests (evaluated at *α* = .017 to correct for multiple comparisons) revealed that the Interpretation students showed a significant gain in score between phase 1 and phase 2 (*t*(46) = 5.916, *p* < .001, *d* = .874, mean gain = 13.6 letters, 95% CI 9.0–18.2), while the Translation and Non-language students did not (*p* = .597 and *p* = .055, respectively). To exclude the possibility that the smaller translation group influenced the result, an ANOVA including only the Interpretation students and the Non-language students was examined. This analysis also revealed an interaction between group and phase (*F*(1,80) = 4.510, *p* = .037). Additionally, to exclude the possibility that the differences in the time elapsed between the phases contributed to the differences in the gain in score, these two factors were tested for a correlation. No correlation was found across the three groups (*r* = −.076, *p* = .472), nor was a correlation found within any of the individual groups (*p*s ≥ .206).

**Table 2:** Memory measures: scores on the four tests of memory by group and phase

	Interpretation students	Translation students	Non-language students
Phase 1			
Letter span score	53.4 (14.8)	55.5 (16.8)	54.0 (17.9)
Matrix span score	43.2 (14.6)	37.0 (13.9)	46.3 (12.3)
Operation span score	42.6 (17.8)	44.9 (20.0)	36.2 (14.3)
Operation span errors	5.9 (3.2)	4.3 (3.0)	6.3 (3.7)
Symmetry span score	17.5 (8.9)	15.2 (10.3)	19.7 (9.7)
Symmetry span errors	3.1 (2.7)	2.6 (2.2)	3.1 (3.4)
Phase 2			
Letter span score	67.0 (17.7)	57.3 (19.0)	59.8 (17.9)
Matrix span score	45.0 (14.4)	36.8 (11.3)	49.7 (11.2)
Operation span score	51.1 (18.4)	48.3 (19.6)	47.6 (15.4)
Operation span errors	4.5 (3.6)	6.3 (4.6)	5.1 (3.1)
Symmetry span score	20.1 (10.3)	15.1 (8.4)	22.6 (10.0)
Symmetry span errors	3.2 (3.8)	3.3 (1.8)	2.5 (2.9)

Values are means with standard deviations in parentheses. The data reported above are for the students who participated longitudinally. The number of items and therefore maximum score on each test were as follows: Letter Span—99; Matrix Span—81; Operation Span—75; Symmetry Span—42

At phase 1, the three groups showed a difference in score on the matrix span task (*F*(2,124) = 4.248, *p* = .016, *η*_*p*_^2^ = .064). Post hoc Tukey tests demonstrated this effect was due to higher scores among the Non-language students than the Translation students (*p* = .012). This difference was also seen in the ANOVA conducted across the phases (*F*(2,89) = 3.350, *p* = .040, *η*_*p*_^2^ = .070), and was again due to higher scores among Non-language students compared to Translation students (*p* = .035). The main effect of phase and the interaction of phase and group were not significant (*p* = .216 and *p* = .580, respectively).

### Working Memory

Analyses on the score on the operation span task revealed no differences among the groups at phase 1 (*p* = .113). Additionally, there were no group differences across the phases (*p* = .335). There was a main effect of phase (*F*(1,89) = 15.191, *p* < .001, *η*_*p*_^2^ = .146), but no interaction between phase and group (*p* = .336). The analysis on the number of errors in the operation span task revealed a group difference at phase 1 (*F*(2,124) = 3.075, *p* = .050, *η*_*p*_^2^ = .047), due to a larger number of errors among the Non-language students than the Translation students (*p* = .040). Across the phases, however, there was no main effect of group (*p* = .710). Further, there was no main effect of phase (*p* = .722); however, there was a trend toward an interaction of phase and group (*F*(2,89) = 2.738, *p* = .070, *η*_*p*_^2^ = .058). Examination of the data revealed a numerical decrease in errors from phase 1 to phase 2 for the Interpretation and Non-language students, but a numerical increase in errors for the students of translation.

Analyses on the score on the symmetry span task revealed no significant effects (*p*s ≥ .128). Similarly, analyses on the number of errors also revealed no effects (*p*s ≥ .421).

### ANT

Three longitudinal participants (a male Interpretation student, a female Translation student, and a female Non-language student) were identified as outliers within their groups on the ANT at phase 1; their data were excluded from all analyses on this task. Two additional participants (female Translation students) were identified as outliers at phase 2; their data were excluded from the longitudinal analyses.

#### Conflict Effect

The conflict effect analyzes the difference between congruent and incongruent trials. Analyses on the phase 1 data revealed group differences in overall accuracy (H(2) = 6.646, *p* = .036) and the accuracy conflict effect (H(2) = 9.698, *p* = .008). These differences were due to higher accuracy on incongruent trials for Translation compared to Non-language students (*p* = .011). The one-way ANOVAs on the RT data at phase 1, however, revealed no effect of group on the overall RTs (*p* = .931) or on the size of the RT conflict effect (*p* = .284). Analyses on the change between phases in overall accuracy and the accuracy conflict effect revealed no differences between the groups (*p* = .238 and *p* = .183, respectively). The ANOVA on RTs across the phases revealed a main effect of trial type (*F*(1,84) = 626.820, *p* < .001, *η*_*p*_^2^ = .882) due to faster responses to congruent trials. The analysis also revealed a main effect of phase (*F*(1,84) = 49.306, *p* < .001, *η*_*p*_^2^ = .370; Table 3), with faster responses at phase 2 than at phase 1. No other effects or interactions were significant (*p*s ≥ .102).

**Table 3:** ANT: mean response times and accuracy by group, phase, and task condition/effect

	Interpretation students		Translation students		Non-language students	
	Response time (ms)	Accuracy (%)	Response time (ms)	Accuracy (%)	Response time (ms)	Accuracy (%)
Phase 1						
Congruent	447 (65)	99.2 (0.9)	501 (133)	99.4 (0.6)	452 (49)	98.7 (1.7)
Incongruent	521 (68)	95.8 (4.6)	553 (115)	98.4 (2.4)	527 (55)	93.0 (6.0)
Conflict effect	73 (21)	3.4 (4.5)	52 (25)	1.0 (2.3)	76 (20)	5.8 (5.4)
Alerting effect	13 (16)	0.0 (2.0)	16 (20)	0.7 (1.2)	7 (14)	0.6 (3.6)
Orienting effect	9 (15)	0.6 (2.5)	1 (27)	0.9 (1.2)	12 (14)	0.8 (2.7)
Phase 2						
Congruent	413 (48)	98.9 (1.2)	429 (39)	99.2 (0.5)	397 (45)	98.2 (2.6)
Incongruent	484 (56)	93.4 (6.1)	492 (37)	98.4 (2.2)	468 (50)	87.7 (10.4)
Conflict effect	71 (23)	5.5 (5.8)	63 (7)	0.8 (2.5)	71 (21)	10.5 (9.2)
Alerting effect	10 (14)	−0.5 (2.3)	9 (11)	0.2 (2.5)	7 (14)	0.4 (3.3)
Orienting effect	9 (14)	0.0 (2.8)	2 (10)	−0.9 (1.8)	8 (13)	0.2 (3.5)

Values in parentheses are standard deviations. The data reported above are for the students who participated longitudinally

#### Alerting Effect

The alerting effect examines the difference between trials cued with the double cue and those with no cue. The size of this effect did not differ between groups at phase 1 in either accuracy (*p* = .931) or RT (*p* = .637). An analysis of the change in the accuracy alerting effect between phases showed no difference between groups (*p* = .905). Further, a three-way ANOVA on RTs including phase and with cue as the trial type showed a main effect of cue (*F*(1,84) = 32.924, *p* < .001, *η*_*p*_^2^ = .282), but no interactions between cue and the phase and group factors (*p*s ≥ .309).

#### Orienting Effect

The orienting effect evaluates the difference between centrally cued and spatially cued trials. As with the alerting effect, the orienting effect was not modulated by group at phase 1 considering both accuracy (*p* = .877) and RTs (*p* = .422). Further, the groups did not differ in the change in the size of the accuracy orienting effect between the phases (*p* = .452). Finally, the three-way ANOVA on RTs with these cues as the trial types revealed a main effect of cue type (*F*(1,84) = 17.419, *p* < .001, *η*_*p*_^2^ = .172), but no interactions of cue type and either phase or group (*p*s ≥ .178).

### Task-Switching Paradigm

One phase 1-only participant (a female Translation student) was identified as an outlier within her group; her data were excluded from all phase 1 analyses on this task. An initial four-way ANOVA including phase, trial type (switch, repetition, single-task), CTI, and group was conducted on the RT data. This analysis revealed a significant effect of CTI length (*F*(1,89) = 589.974, *p* < .001, *η*_*p*_^2^ = .869), due to longer responses to the short CTI; however, there were no interactions between the group and CTI factors (*p*s ≥ .103). Thus, to simplify the analyses reported below, the two CTI lengths were collapsed.

#### Switching Costs

Switching costs represent the difference in performance on switch trials and repetition trials in the mixed-task block. Analyses on the accuracy data at phase 1 revealed no group differences on mixed-block accuracy (*p* = .097) or on the accuracy switching cost (*p* = .965). Similarly, there were no group differences in mixed-block RTs (*p* = .237) or RT switching cost (*p* = .467) at phase 1. An analysis of the change in mixed-block accuracy between phase 1 and phase 2 revealed a group difference (H(2) = 7.056, *p* = .029), with post hoc analyses revealing only a marginally larger decrease among Translation students than Interpretation students (*T* = 21.081, *z* = 2.275, *p* = .069). A similar analysis on the change in accuracy switching cost, however, revealed no group difference (*p* = .103). The three-way ANOVA on RTs across phases evidenced main effects of phase (*F*(1,89) = 53.132, *p* < .001, *η*_*p*_^2^ = .374) and trial type (*F*(1,89) = 180.361, *p* < .001, *η*_*p*_^2^ = .670). These were due to faster responses at phase 2 compared to phase 1 and to repetition trials compared to switch trials. Additionally, these factors showed a significant interaction (*F*(1,89) = 13.650, *p* < .001, *η*_*p*_^2^ = .133; Table 4), revealing that switching costs also decreased between phase 1 and phase 2. No effects with group were significant (*p*s ≥ .333).

**Table 4:** Task-switching paradigm: mean response times and accuracy by group, phase, and task condition/effect

	Interpretation students		Translation students		Non-language students	
	Response time (ms)	Accuracy (%)	Response time (ms)	Accuracy (%)	Response time (ms)	Accuracy (%)
Phase 1						
Single task	469 (87)	98.1 (2.5)	514 (98)	99.3 (1.5)	487 (52)	97.7 (2.5)
Repetition	773 (202)	97.4 (2.2)	811 (222)	98.0 (2.3)	806 (169)	96.5 (2.3)
Switch	929 (269)	95.0 (4.5)	990 (270)	95.1 (3.8)	960 (207)	94.0 (4.1)
Mixing cost	304 (156)	0.7 (2.6)	297 (158)	1.4 (2.6)	320 (158)	1.2 (2.5)
Switching cost	155 (101)	2.4 (3.8)	179 (95)	2.9 (2.6)	154 (85)	2.5 (3.4)
Phase 2						
Single task	423 (66)	98.1 (2.7)	422 (60)	97.6 (3.8)	445 (71)	97.6 (2.4)
Repetition	666 (187)	97.3 (2.4)	640 (196)	96.4 (4.7)	673 (130)	95.9 (2.5)
Switch	797 (250)	94.5 (4.8)	787 (253)	89.4 (7.7)	778 (150)	91.9 (4.6)
Mixing cost	242 (141)	0.9 (2.5)	218 (146)	1.2 (2.4)	228 (107)	1.7 (3.8)
Switching cost	131 (99)	2.7 (3.8)	147 (98)	7.0 (5.9)	105 (68)	4.0 (4.1)

Values in parentheses are standard deviations. The data reported above are for the students who participated longitudinally

#### Mixing Costs

Mixing costs represent the difference in performance on repetition trials in the mixed-task block and trials in the single-task blocks. The groups did not differ in accuracy on single-task trials at phase 1 (*p* = .378), though the difference in accuracy mixing cost was marginal (H(2) = 5.982, *p* = .050). Further, at phase 1 there were no group differences in RTs from the single-task block (*p* = .118) or the size of the mixing cost (*p* = .528). Analyses on the change between phase 1 and phase 2 in accuracy on single-task trials and the accuracy mixing cost revealed no group differences (*p* = .264 and *p* = .752, respectively). The three-way ANOVA on RTs with phase, trial type, and group revealed main effects of phase (*F*(1,89) = 67.759, *p* < .001, *η*_*p*_^2^ = .432) and trial type (*F*(1,89) = 281.760, *p* < .001, *η*_*p*_^2^ = .760). These were due to faster responses at phase 2 and to single-task trials. These two factors also interacted (*F*(1,89) = 16.708, *p* < .001, *η*_*p*_^2^ = .158; Table 4), revealing a decrease in mixing cost between phase 1 and phase 2. No effects with group were significant (*p*s ≥ .237).

## Discussion

This study investigated the provenance of advantages in memory and executive functioning previously ascribed to professional interpreters through a longitudinal study of students of interpretation and control groups with and without similar language expertise. At the start of training, no advantages in memory or executive functioning were found for the students of interpretation. A specific effect of training in SI, however, was evident in verbal short-term memory performance, where the students of interpretation showed a large gain. Additionally, improvements in performance between phase 1 and phase 2 were seen across the tasks and groups.

The present data showed no advantage among the students of interpretation on any measure at phase 1. Thus, it appears that the advantages in memory and executive functioning seen among professional interpreters in previous studies are not due to inherent differences between individuals who pursue simultaneous interpretation and those who do not. These findings are in sync with a recent study that examined students of consecutive interpretation, students of translation, and students of English culture (Dong and Liu 2016). That study found no differences on a number Stroop task, a task-switching paradigm, or 2-back task at the start of training. Differences at phase 1 in the present study, however, were seen between the Translation students and the Non-language students. These differences could potentially be related to their diverging educational backgrounds; however, a further discussion of these effects is beyond the scope of this study.

The present findings did, however, suggest that specific abilities are enhanced through training in simultaneous interpretation. In particular, the students of interpretation showed an increase in verbal short-term memory, which was not evident in the students of translation and the Non-language students. It appears then that the advantages in verbal short-term memory seen among professional interpreters (e.g., Babcock and Vallesi 2017; Bajo et al. 2000; Christoffels et al. 2006; Padilla et al. 1995; Stavrakaki et al. 2012) are likely the result of training in simultaneous interpretation, rather than inherent characteristics. Notably, this enhancement was not evident in the spatial domain, but was limited to the verbal domain. That SI would target verbal memory is extremely plausible as it is likely employed during SI to store content and rehearse pre-output reformulations.

These results are the first to show an improvement in short-term memory associated with training in SI as the two other longitudinal studies of SI training and memory did not include short-term memory measures (Chmiel 2016; Macnamara and Conway 2014). Those studies did, however, include measures of working memory. Macnamara and Conway (2014) found improvements in number-letter sequencing and backward digit span, but no improvement in reading span or operation span, while Chmiel (2016) found an improvement on reading span in the second language. It is interesting to note that while there was no specific advantage of SI training on performance in the operation span task in the present study, there was an improvement in performance across the groups. This is in line with the results from Chmiel (2016) and highlights the importance of including control groups in longitudinal designs. However, results from that study and the present study are discordant with those found by Macnamara and Conway (2014). One possible explanation for this discrepancy is a potential difference in the specific measure considered. The former studies used the number of recalled items as the measure of interest, while the latter reported values between 0 and 1, but did not indicate their formulation. If these values were a composite of the items recalled and errors committed, this may explain the different findings.

Though no SI training-modulated effects were apparent on the executive functioning tasks, there were improvements across the groups in the overall response time on the ANT and task-switching paradigm, as well as in the size of the switching and mixing costs. Macnamara and Conway (2014) also considered a measure of task-switching, though using a different paradigm, and similarly found an enhancement in performance at the second testing. Our improvement in switching cost is in accordance with this finding, but suggests that it may be due to a learning effect, not SI training, which could not be disentangled without a control group.

Further, these general improvements on executive functioning tasks are largely concordant with a recent longitudinal study of consecutive interpretation students, translation students, and English culture students (Dong and Liu 2016). That study found decreased overall response times on a number Stroop task, a task-switching paradigm, and a visuo-spatial 2-back task at the second time point across the groups. Unlike our results, however, an improvement in mixing cost was not seen. This may be due to their use of univalent rather than bivalent stimuli, which likely made the task easier (indeed, their mixing costs were sizably smaller than ours; ∼120 vs. ∼310 ms at phase 1 and ∼105 vs. ∼235 ms at phase 2) and allowed for close to ceiling performance even at the first testing.

Finally, an improvement in switching costs specific to the consecutive interpretation students was also found. The switching and mixing cost results in comparison with the previously evidenced simultaneous interpreter advantage on mixing cost, but not switching cost (Babcock and Vallesi 2017; Becker et al. 2016), may point to the specificity of enhancements to the training/experience received. Simultaneous interpretation and consecutive interpretation (CI) are similar in many aspects; however, they differ in the timing of the delivery of the interpreted content. As discussed in the “Introduction,” during SI the output overlaps with the input, requiring simultaneous use and maintenance of the two languages. The processes recruited for this maintenance are likely those measured by the mixing cost. Conversely, during CI, the interpreted output is delivered after the input in pauses for that purpose, requiring the consecutive interpreter to switch back and forth between the languages. Therefore, it may be conjectured that these individuals exercise their switching abilities. Future studies that are able to adequately separate the two types of interpretation would be highly informative about the specificity of enhancements.

Our results also hint to some differential effects based on the training experience. Two measures showed training-modulated effects specific to translation training. On the verbal working memory task, the Interpretation and Non-language students showed a pattern of decreasing errors, while the Translation students showed the opposite pattern. Though these results should be regarded with caution as the interaction was marginal and the post hoc analyses on this measure were not significant. Additionally, accuracy in the mixed-task block of the task-switching paradigm showed an effect of translation training, with the Translation students showing a larger decrease in accuracy than the Interpretation students. While these two results may be suggestive of a translation training-specific effect, any conclusions would be premature given the small number of Translation students who participated longitudinally. Additionally, there are no studies of professional translators that have suggested such effects. Thus we leave a full discussion of these effects for future studies on professional translators and translation students that replicate the findings.

Finally, though the present findings suggest that enhanced verbal short-term memory in professional interpreters is likely the result of initial training in SI, they are agnostic on the provenance of the previously found advantages in spatial short-term memory, verbal working memory, and sustained control (e.g., Babcock and Vallesi 2017; Bajo et al. 2000; Christoffels et al. 2006). It may then be that these advantages emerge after further experience with interpretation rather than after the 2-year training period. In this case, the selective enhancement of verbal short-term memory during the training period may suggest that this benefit is a prerequisite for the advantages seen in working memory and sustained control. Alternatively, a case could be made that the student groups are at peak performance and therefore any group differences are obscured. These differences rather would emerge as the population ages at which point interpretation would provide a protective benefit similar to that posited for bilingualism (e.g., Bialystok et al. 2007; Luk et al. 2011). Future longitudinal studies which follow interpreters at the start of their careers may be able to disentangle these alternatives as well as add to our understanding of the separate roles of SI training and SI experience. These studies should take care to select appropriate control populations. We attempted to do this with the inclusion of students of translation, though with a small number of (returning) participants. Thus, an examination of students of interpretation compared to a larger sample of students of translation is advisable. Additionally, a wider array of measures could be considered as interpreters may show enhancements in executive functions and cognitive abilities not investigated in the present study. Finally, future longitudinal studies that seek to understand the relationship between cognitive abilities and interpretation abilities, similar to Macnamara and Conway (2016), would increase our understanding of this demanding skill.

The current results also make contributions to the growing literature on the cognitive effects of executive function training. Though a multitude of studies have investigated the potential to improve general cognition through such training, consistent evidence for an effect has been limited (e.g., Melby-Lervåg and Hulme 2013; Shipstead et al. 2012). The majority of these studies, however, have seen participants repeating a narrowly focused task in a laboratory setting, a situation that does not closely resemble our multifaceted world experience. The present study, on the other hand, examined the effects of a cognitively demanding training in an ecologically valid setting and found evidence of an enhancement in memory. These findings strengthen the possibility that holistic life experiences lead to cognitive enhancements. Future studies may wish to use interpretation training and other cognitively demanding life experiences, such as pilot training and air traffic controller training (e.g., Arbula et al. 2016), as ecologically valid models for brain training.

## Conclusion

The present study represents an initial effort to understand the evolution of cognitive processes in individuals learning to perform simultaneous interpretation. The results suggested that interpreters do not possess inherent advantages in memory and executive functioning, but rather that these advantages emerge through SI training and later experience. Further, they provide evidence that ecologically valid training does lead to specific enhancements in short-term memory. Future longitudinal studies of training and initial professional experience in simultaneous interpretation will contribute greatly to our understanding of the processes used during this highly demanding skill. Additionally, such studies will increase our general understanding of skill learning in adulthood and provide a model of ecologically valid brain training.

### Electronic Supplementary Material

Below is the link to the electronic supplementary material.

ESM 1 (DOCX 15 kb)

## Appendix: Functionally Fluent Questionnaire

### English Version

Please list all the foreign languages you have studied or know. For each language please answer the following questions with that language in mind.

1. Do you know the A1, A2, B1 etc. system of classifying language levels? If yes, what is your level in this language?
2. Could you discuss a topic in which you are not an expert, such as politics, in this language?
3. Can you understand news programs in this language?
4. Could you read a novel or short story for pleasure in this language?
5. Could you tell a story about events in the past, present, and future to a group of people in this language?
6. Could you write a letter in this language to a friend about an important event in your life and how it affected you?
7. Would you be able to understand an announcement about a canceled train in this language and follow the directions given about where to refund or change your ticket?
8. Could you write an essay in this language on a work of literature?
9. Could you understand a textbook passage on your field of study in this language?

### Italian version

Per favore elenca le lingue straniere che hai studiato o che conosci. Per ogni lingua, per favore rispondi alle seguenti domande riferendoti a quella lingua.

1. Conosci il sistema di classificazione A1, A2, B2, ecc. che indica il livello di conoscenza di una lingua? Se si, quale e’ il tuo livello in questa lingua?
2. Potresti parlare di un tema del quale non sei esperto, come la politica, in questa lingua?
3. Potresti comprendere un telegiornale in questa lingua?
4. Potresti leggere un romanzo o un breve racconto per passatempo in questa lingua?
5. Potresti raccontare una storia su un evento del passato, uno del presente e uno del futuro a un gruppo di persone in questa lingua?
6. Potresti scrivere una lettera in questa lingua ad un amico riguardo un evento importante della tua vita e di come ti ha influenzato?
7. Saresti capace di comprendere un annuncio riguardo un treno cancellato in questa lingua e seguire le istruzioni date su dove farti rimborsare o cambiare il biglietto?
8. Potresti scrivere un saggio in questa lingua su un brano di letteratura?
9. Potresti comprendere un passaggio di un libro di testo nel tuo campo di interesse in questa lingua?

## Footnotes

1 Though some Triennale degrees in Languages introduce the basics of Translation and Interpretation, they do not cover the skill of simultaneous interpretation. Thus, all participants were unskilled in SI at the start of the study.

2 All students were invited to participate longitudinally. Individuals who chose not to complete testing at phase 2 by and large cited living in a different city as their reason for not returning. However, to check that there was no selection bias for only the best students returning, we compared the background variables (age, years of education, and SES) for the returners and non-returners within each group. There were no differences between returners and non-returners on any of these variables within any of the three groups (all *p*s ≥ .155). Further, we also examined the language-related variables (language ability ratings, switching frequency, and number of languages used, see *Language history questionnaire* for details on these measures) within the Interpretation and Translation students. Among the Translation students, there were no differences between the returners and non-returners on any of these measures (all *p*s ≥ .105). Among the Interpretation students, there were no differences in the switching frequency and number of languages used (*p*s ≥ .264). The non-returners did report higher self-ratings across the language abilities than the returners (*p*s ≤ .061). Given that this bias is toward the non-returners, it does not seem to be the case that only the best students completed phase 2.

3 The higher attrition rate among Translation students was not due to failure to complete the Master’s program, but rather to their differing graduation schedule which resulted in only ten of them being available for retesting at phase 2.
