# Supplementary material for: Short-Term Memory Improvement After Simultaneous Interpretation Training
Source: J Cogn Enhanc. 2017 Feb 27;1(3):254–67. doi: 10.1007/s41465-017-0011-x (PMC7089711; doi:10.1007/s41465-017-0011-x)
Supplement: Supplementary file 1 — (DOCX 15 kb) [file 41465_2017_11_MOESM1_ESM.docx]

Supplementary Table 1: Biographical and language characteristics of the original groups

|  | Interpretation students (N=55) | Translation students (N=21) | Non-language students (N=51) |
| --- | --- | --- | --- |
| Age at Phase 1 (in years) | 22.7 (1.7) | 23.3 (2.6) | 23.1 (1.8) |
| Years of education at Phase 1 | 16.3 (1.0) | 16.3 (1.0) | 16.5 (1.8) |
| Mother’s years of education^a^ | 13.6 (3.6) | 13.6 (2.8) | 12.3 (3.1) |
| Phase 1 averaged reading level^b,c^ | 4.3 (0.5) | 4.2 (0.5) |  |
| Phase 1 averaged writing level^b,c^ | 3.7 (0.5) | 3.6 (0.6) |  |
| Phase 1 averaged speaking level^b,c^ | 3.9 (0.5) | 3.4 (0.6) |  |
| Phase 1 averaged understanding level^b,c^ | 4.2 (0.4) | 4.1 (0.6) |  |

| Switching frequency at home/with friends^d^ | 3.4 (1.3) | 3.4 (1.2) |  |
| --- | --- | --- | --- |
| Switching frequency at school^d^ | 4.0 (1.0) | 3.9 (1.0) |  |
| Number of languages used at home/with friends^e^ | 2.9 (1.1) | 2.4 (1.0) |  |
| Number of languages used at school^e^ | 3.7 (0.8) | 3.5 (0.6) |  |

Note: Values in parentheses are standard deviations. ^a^Data were not available for four Non-language students and one Interpretation student. ^b^These values were averaged across the two or three languages each participant studied as part of their Master’s program. ^c^Data were not available for eight Interpretation students. ^d^Data were not available for eleven Interpretation students. ^e^Data were not available for five Interpretation students.
